# Effects of Budesonide on Coronavirus-Associated Receptor and Immune-Mediator Expression in Human Lung Microvascular Endothelial Cells

**DOI:** 10.3390/medicina62071347

**Published:** 2026-07-12

**Authors:** Izabela Ławska, Adrian Bekier, Maciej Chałubiński, Aleksandra Wardzyńska

**Affiliations:** Department of Immunology and Allergy, Medical University of Lodz, 92-213 Lodz, Poland

**Keywords:** budesonide, coronavirus entry receptors, microvascular endothelial cells

## Abstract

*Background and Objectives*: Inhaled corticosteroids exert broad immunomodulatory effects in patients with chronic airway diseases. However, their direct impact on pulmonary endothelial immune responses and coronavirus-associated receptor expression remains unclear. This study investigated the effects of budesonide on immune responses and the expression of coronavirus entry receptors in human lung microvascular endothelial cells (HMVEC-L). *Materials and Methods:* HMVEC-L cells were exposed to budesonide (1 ng/mL), a non-cytotoxic concentration selected based on cell viability assays. The mRNA expression of angiotensin-converting enzyme 2 (ACE2), dipeptidyl peptidase-4 (DPP4), aminopeptidase N (AP-N), intercellular adhesion molecule 1 (ICAM-1), interferon beta (IFN-β), RANTES/CCL5, and interleukin-8 (IL-8/CXCL8) was analyzed using quantitative RT-PCR. The surface expression of ACE2, DPP4, AP-N, and ICAM-1 was assessed using flow cytometry. Secreted IL-8 concentration was measured using ELISA. *Results:* Budesonide significantly reduced AP-N and DPP4 mRNA expression, accompanied by a decrease in the surface expression of both receptors. ACE2 mRNA expression was transiently reduced, whereas ACE2 surface expression was modestly increased by approximately 5% at 72 h. Budesonide also reduced early ICAM-1 mRNA expression but increased its surface expression at the later time point. Budesonide significantly reduced RANTES/CCL5 and IL-8/CXCL8 mRNA expression, with a corresponding decrease in secreted IL-8 concentration, whereas IFN-β mRNA expression showed a non-significant statistical decrease. *Conclusions:* Budesonide directly modulates pulmonary endothelial immune responses and coronavirus-associated receptor expression. These findings indicate that budesonide modulates the expression of coronavirus-associated receptors and basal antiviral and inflammatory mediators in HMVEC-L cells. Because viral binding, entry, replication, and infection were not assessed, these results should be interpreted as evidence of receptor and immune-mediator modulation rather than as demonstrating altered coronavirus susceptibility.

## 1. Introduction

Chronic inflammatory diseases of the respiratory tract, including asthma and chronic obstructive pulmonary disease (COPD), result from complex interactions between environmental exposure, viral infections, and dysregulated immune responses [1]. Inhaled corticosteroids (ICS) are the mainstay of anti-inflammatory treatment for these diseases and exert broad immunomodulatory effects that extend beyond the suppression of airway inflammation [1,2,3].

Recent evidence indicates that corticosteroids may modulate the host-cell entry factors involved in coronavirus infection. Inhaled corticosteroids have been shown to alter the expression of angiotensin-converting enzyme 2 (ACE2), a key host receptor for SARS-CoV-2, primarily in airway epithelial cells [2,3]. However, the effects of corticosteroids on other coronavirus-associated receptors remain poorly understood.

The pulmonary vascular endothelium is an active component of the respiratory immune microenvironment, contributing to inflammatory signaling, leukocyte recruitment, and vascular homeostasis [4,5]. In addition to its immunoregulatory role, the pulmonary endothelium expresses several molecules implicated in coronavirus entry, including ACE2, dipeptidyl peptidase-4 (DPP4), and aminopeptidase N (AP-N), which serve as entry receptors for SARS-CoV-2, SARS-CoV, MERS-CoV, and human coronavirus 229E, respectively [5,6].

Our previous study demonstrated that human lung microvascular endothelial cells express ACE2, DPP4, and AP-N, and that their expression can be regulated by inflammatory stimuli [6]. Despite increasing interest in the immunomodulatory effects of inhaled corticosteroids in respiratory viral diseases, their direct effects on pulmonary endothelial immune responses and coronavirus-associated receptor expression remain unknown.

Therefore, this study aimed to investigate the direct effects of budesonide on immune responses and coronavirus entry receptor expression in human lung microvascular endothelial cells (HMVEC-L).

## 2. Materials and Methods

### 2.1. Cells

Human lung microvascular endothelial cells (HMVEC-L) from two different donors (CC-2527, Lonza, Walkersville, MD, USA) were cultured in Endothelial Cell Basal Medium 2 (C-2221, PromoCell, Heidelberg, Germany), supplemented with Endothelial Growth Medium-2 MV (C-39221, PromoCell, Heidelberg, Germany). The cells were cultured in an incubator at 37 °C with 5% CO2. Upon reaching 80–90% confluence, the cells were trypsinized with 0.25% trypsin and 0.02% ethylenediaminetetraacetic acid (EDTA) (59417C, Sigma-Aldrich, St. Louis, MO, USA) and neutralized using Trypsin Neutralizing Solution (CC-5002, Lonza, Walkersville, MD, USA). The cells were initially seeded at a density of 2.0 × 10^5^ cells/well in 24-well plates. After 24 h of incubation under standard conditions, the cells were ready for experimentation.

### 2.2. Budesonide Stimulation: Experimental Conditions and Effects on HMVEC-L Viability

**Figure 1:**
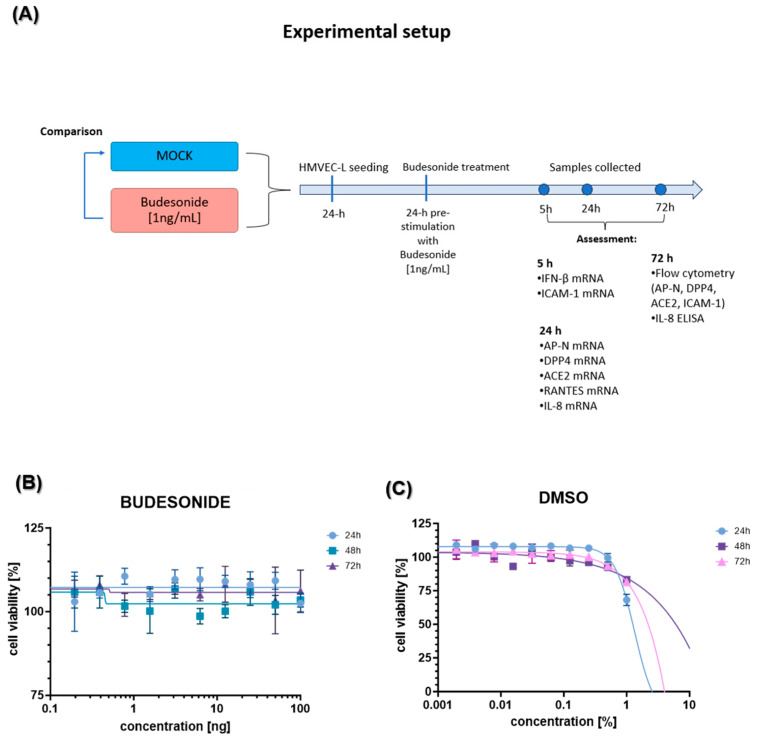
Experimental setup and selection of budesonide concentration. Selection of a non-cytotoxic budesonide concentration and experimental design in human lung microvascular endothelial cells (HMVEC-L). (**A**) Schematic representation of the experimental setup used in the study. Human lung microvascular endothelial cells (HMVEC-L) were stimulated with budesonide (1 ng/mL). HMVEC-L cells were pre-exposed to budesonide (1 ng/mL) for 24 h, after which samples were collected at the indicated time points depending on the analyzed endpoint. Budesonide remained present in the culture medium throughout the experiment. (**B**) Cell viability of HMVEC-L after exposure to increasing concentrations of budesonide for 24 h, 48 h, and 72 h. (**C**) Cell viability of HMVEC-L after exposure to increasing concentrations of DMSO for 24 h, 48 h, and 72 h. Data are representative of at least four independent experiments presented as means ± SEM; *n* = 4–10.

HMVEC-L cells were exposed to budesonide (Sigma-Aldrich, St. Louis, MO, USA) at a final concentration of 1 ng/mL. Budesonide was dissolved in dimethyl sulfoxide (DMSO), and the final DMSO concentration corresponding to the working budesonide dilution was 0.00001%. Based on cell viability assays, 1 ng/mL was selected for all subsequent experiments as a non-cytotoxic concentration (Figure 1B). HMVEC-L cells were pre-exposed to budesonide for 24 h. After this pre-exposure period, samples were collected after an additional 5, 24, or 72 h, depending on the analyzed endpoint (Figure 1A). Budesonide remained present in the culture medium during both the pre-exposure period and the subsequent endpoint-specific incubation. Early transcriptional responses (IFN-β and ICAM-1) were analyzed after 5 h, AP-N, DPP4, ACE2, RANTES/CCL5, and IL-8/CXCL8 mRNA expression after 24 h, whereas surface receptor expression and IL-8 secretion were evaluated after 72 h. Untreated HMVEC-L cells were used as the control group in the main molecular, flow-cytometric, and ELISA experiments. The potential effect of the vehicle was assessed separately in the viability assay using DMSO dilutions corresponding to those used for budesonide preparation. The final DMSO concentration corresponding to 1 ng/mL budesonide did not affect HMVEC-L viability at any analyzed time point (Figure 1C). Based on these findings, a budesonide concentration of 1 ng/mL was chosen for all subsequent experiments.

### 2.3. Cell Viability Assay

The cytotoxic effects of budesonide and its vehicle (DMSO) on human lung microvascular endothelial cells (HMVEC-L) were assessed using a resazurin-based viability assay. HMVEC-L cells were seeded in 96-well plates at a density of 2 × 104 cells/well and cultured until confluence. The cells were then exposed to increasing concentrations of budesonide (0.1–100 ng/mL) or the corresponding concentrations of DMSO (0.001–10%) for 24, 48, and 72 h at 37 °C in a humidified atmosphere containing 5% CO2. Cell viability was determined using resazurin (Sigma-Aldrich, Cat. No. R7017). Viable cells with active mitochondrial metabolism reduce non-fluorescent resazurin to resorufin. Resazurin solution (0.2 mg/mL in sterile PBS) was added to each well (20 μL per well) 4 h before the end of incubation and was maintained at 37 °C and 5% CO_2_. Resazurin reduction was measured using a Multiskan GO microplate spectrophotometer (Thermo Fisher Scientific, Vantaa, Finland) in absorbance mode. Absorbance was read at 570 nm, with 600 nm used as the reference wavelength. The background signal from the medium-only wells was then subtracted. The results are presented as the percentage (%) of control cells at each time point.

### 2.4. Isolation of mRNA and Real-Time Polymerase Chain Reaction (RT-PCR)

mRNA was extracted from HMVEC-L cells using the ReliaPrep RNA Cell Miniprep System (Z6012, Promega, Madison, WI, USA) according to the manufacturer’s instructions. Subsequently, cDNA was synthesized using the RevertAid H Minus First Strand cDNA Synthesis Kit (K1632, Thermo Fisher Scientific, Waltham, MA, USA). Gene expression was analyzed by quantitative reverse transcription PCR (qRT-PCR) using TaqMan Gene Expression Assays and TaqMan^®^ Universal PCR Master Mix (Applied Biosystems, Thermo Fisher Scientific, Cat. No. 4304437). The following TaqMan Gene Expression Assays were used: ACE2, Hs01085333_m1; ANPEP/CD13, Hs00174265_m1; DPP4/CD26, Hs00897386_m1; ICAM1, Hs00164932_m1; IFNB1, Hs01077958_s1; CCL5/RANTES, Hs00982282_m1; CXCL8/IL8, Hs00174103_m1; and GAPDH, Hs02786624_g1. For each reaction, 1 µL of cDNA was diluted with 3.5 µL of nuclease-free water and combined with 5.5 µL of reaction mix containing 0.5 µL of the corresponding TaqMan assay and 5 µL of TaqMan^®^ Universal PCR Master Mix. qRT-PCR was performed using the StepOnePlus Real-Time PCR System (Applied Biosystems, Foster City, CA, USA) under the following cycling conditions: 50 °C for 2 min and 95 °C for 10 min, followed by 45 amplification cycles of 95 °C for 15 s and 60 °C for 1 min. Relative gene expression was normalized to GAPDH and calculated using the 2^−ΔΔCt^ method, with untreated control cells used as the calibrator. GAPDH was selected as the reference gene because its Ct values remained stable across untreated and budesonide-treated samples under the experimental conditions used in this study.

### 2.5. Cytokine Concentration

Cytokine concentrations were measured using enzyme-linked immunosorbent assays (ELISA) specific for each protein, following the manufacturer’s instructions. IL-8/CXCL8 (Human IL-8/CXCL8 DuoSet ELISA (R&D Systems, Minneapolis, MN, USA), Cat. no DY208, Assay range—31.3–2000 pg/mL). The test was purchased from R&D Systems (Minneapolis, MN, USA).

### 2.6. Analysis of ACE2, DPP4, AP-N and ICAM-1 Surface Expression on HMVEC-L by Flow Cytometry

Protein surface expression in HMVEC-L cells was assessed using flow cytometry 72 h after budesonide stimulation. Before staining, cells were detached using trypsin-EDTA and washed with HEPES-buffered saline. The cells were then blocked with 1% bovine serum albumin (BSA; A790650G, Sigma-Aldrich, St. Louis, MO, USA) in PBS and incubated with specific antibodies or corresponding isotype control antibodies for 30 min on ice, as described in Supplementary Table S1. After incubation, the cells were washed with HEPES-buffered saline. Data were acquired using a BD LSRFortessa flow cytometer (BD Biosciences, San Jose, CA, USA)and analyzed using BD FACSDiva software (version 8.0, BD Biosciences, San Jose, CA, USA). Cell populations were first selected based on FSC-A/SSC-A characteristics, and doublets were excluded using FSC-A/FSC-H parameters. Surface marker expression was then evaluated within the gated single-cell population. Isotype-matched controls were used to define non-specific background staining. Surface expression was quantified as mean fluorescence intensity (MFI) and normalized to untreated control cells. Because the staining profiles showed continuous fluorescence distributions rather than clearly separated positive and negative populations, MFI rather than percentage of positive cells was used for quantitative analysis. A representative gating strategy is shown in Supplementary Figure S1. Raw and normalized MFI values used for flow-cytometric analysis are provided in Supplementary Table S2.

### 2.7. Statistical Analysis

Results are presented as mean ± standard error of the mean (SEM) from independent experiments. HMVEC-L cells derived from two independent donors were used in this study. The reported *n* values refer to independent experimental replicates performed on separate cell cultures and stimulations, not to technical replicates or to the number of donors. No technical replicates were included in the qRT-PCR, ELISA, or flow-cytometric datasets. Therefore, each data point represents one independent experiment. Because of the limited sample size, normality testing was not used as a criterion for selecting parametric tests, and the non-parametric Mann–Whitney U-test was applied for comparisons between two independent groups. Statistical significance was set at *p* < 0.05. Statistical analyses were performed using the GraphPad Prism software, version 10.0 (GraphPad Software, La Jolla, CA, USA).

## 3. Results

### 3.1. Budesonide Modulates Expression of Coronavirus Entry Receptors and ICAM-1 in Lung Endothelial Cells

**Figure 2:**
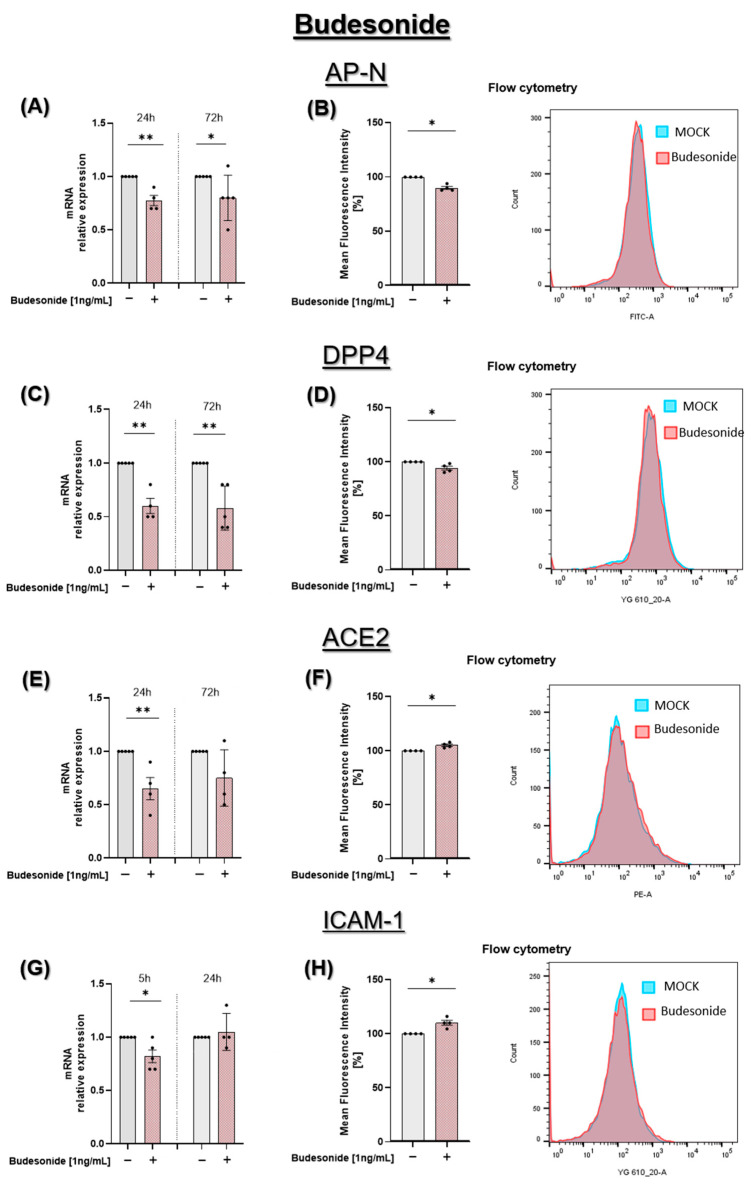
Budesonide modulates expression of coronavirus entry receptors and ICAM-1 of HMVEC-L. Relative mRNA expression of aminopeptidase N (AP-N) (**A**), dipeptidyl peptidase-4 (DPP4) (**C**), angiotensin-converting enzyme 2 (ACE2) (**E**), and intercellular adhesion molecule 1 (ICAM-1) (**G**) was assessed in HMVEC-L exposed to budesonide (1 ng/mL). AP-N, DPP4, ACE2 mRNA expression was analyzed at 24 h and 72 h and ICAM-1 mRNA expression at 5 h and 24 h. Surface expression of AP-N (**B**), DPP4 (**D**), ACE2 (**F**), and ICAM-1 (**H**) was assessed by flow cytometry at 72 h. Relative mRNA expression was normalized to control cells. The Mann–Whitney U-test was used to analyze differences between the two groups. Data are representative of at least four independent experiments and are presented as means ± SEM; * *p* < 0.05; ** *p* < 0.01. (Exact sample sizes and *p*-values are provided in Supplementary Table S3).

Budesonide exposure resulted in a significant reduction in AP-N mRNA expression at both 24 and 72 h compared to that in control cells. At 24 h, AP-N mRNA expression was reduced to approximately 0.78-fold of control, corresponding to an approximately 22% reduction (*p* < 0.01), whereas at 72 h, the reduction was less pronounced, with expression reduced to approximately 0.80-fold of control, corresponding to an approximately 20% reduction (*p* < 0.05) (Figure 2A). Consistent with these transcriptional changes, AP-N surface expression, assessed by flow cytometry at 72 h, was modestly reduced by approximately 10% in budesonide-exposed cells compared to that in control cells (*p* < 0.05) (Figure 2B).

Similarly, DPP4 mRNA expression was significantly reduced following budesonide exposure at 24 and 72 h compared to that in control cells. At 24 h, DPP4 mRNA expression was reduced to approximately 0.60-fold of control (*p* < 0.01), whereas at 72 h, the reduction was similar, with expression reduced to approximately 0.58-fold of control (*p* < 0.01) (Figure 2C). At the protein level, DPP4 surface expression assessed at 72 h was modestly but significantly reduced by approximately 6% in budesonide-exposed cells compared to that in control cells (*p* < 0.05) (Figure 2D).

Budesonide treatment significantly affected ACE2 mRNA expression, but only at 24 h. At this time point, ACE2 mRNA expression was reduced to approximately 0.65-fold of control (*p* < 0.01) (Figure 2E), whereas no significant difference was observed at 72 h. In contrast to the transcriptional changes, ACE2 surface expression assessed at 72 h was modestly increased by approximately 5% in budesonide-exposed cells compared to that in control cells (*p* < 0.05) (Figure 2F).

Budesonide exposure had a time-dependent effect on ICAM-1 expression. ICAM-1 mRNA expression was significantly reduced to approximately 0.82-fold of control at 5 h (*p* < 0.05) (Figure 2G). At 24 h, ICAM-1 mRNA expression showed a slight increase relative to that in the control cells; however, this change was not statistically significant. Despite early transcriptional suppression, ICAM-1 surface expression, assessed by flow cytometry at 72 h, modestly increased by approximately 10% in budesonide-exposed cells compared to control cells (*p* < 0.05) (Figure 2H).

### 3.2. Budesonide Modulates Basal Antiviral and Inflammatory Responses in HMVEC-L

**Figure 3:**
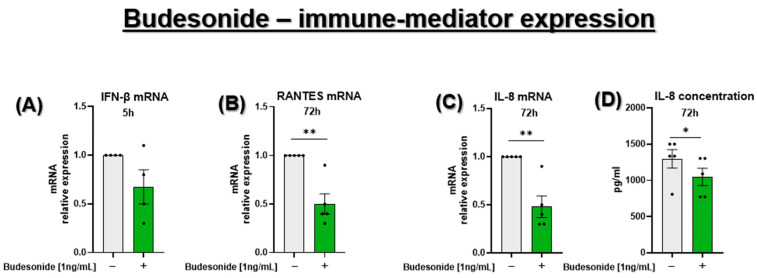
Budesonide modulates basal immune-mediator expression in HMVEC-L. Relative mRNA expression of IFN-β (**A**), RANTES/CCL5 (**B**), and IL-8/CXCL8 (**C**), and secreted IL-8 concentration (**D**), were assessed in human lung microvascular endothelial cells under basal conditions following budesonide exposure (1 ng/mL). Data are presented as means ± SEM; each dot represents one independent experiment. Relative mRNA expression was normalized to control cells. The Mann–Whitney U-test was used to analyze differences between the two groups. Data are representative of at least four independent experiments and are presented as means ± SEM; * *p* < 0.05; ** *p* < 0.01. Exact sample sizes and *p*-values are provided in Supplementary Table S3.

IFN-β mRNA expression was lower in budesonide-treated cells than in control cells. However, this difference did not reach statistical significance (Figure 3A; Supplementary Table S3). Next, RANTES/CCL5 mRNA expression in budesonide-treated cells was reduced to approximately 50% of control values (*p* < 0.01) (Figure 3B).

Subsequently, IL-8/CXCL8 mRNA expression in budesonide-treated cells was reduced to approximately 48% of control values (*p* < 0.01) (Figure 3C). This was accompanied by a decrease in secreted IL-8 concentration (1048.6 ± 118.9 vs. 1295.6 ± 127.5 pg/mL, budesonide vs. control cells, *p* < 0.05) (Figure 3D).

## 4. Discussion

The present study shows that budesonide directly modulates the pulmonary endothelial phenotype of human lung microvascular endothelial cells, affecting both coronavirus-associated receptor expression and basal antiviral and inflammatory mediator expression in these cells. This is the first study to specifically evaluate the direct effects of budesonide on the pulmonary endothelial expression of ACE2, DPP4, and AP-N, together with selected immune mediators relevant to antiviral defense. These findings indicate that budesonide reduces AP-N and DPP4 mRNA expression, accompanied by a decrease in the surface expression of both receptors. Budesonide also reduced ACE2 mRNA expression, increased its surface expression, and modulated the basal endothelial immune-mediator profile by reducing IFN-β, RANTES, and IL-8. Although statistically significant, the observed changes in surface receptor expression were modest, ranging approximately from 5% to 10%. Therefore, their biological significance cannot be inferred from expression-based analysis alone. These findings should be interpreted as evidence of budesonide-mediated modulation of the endothelial surface phenotype rather than as proof of altered receptor function, ligand binding, viral entry, or infection susceptibility. Functional studies assessing receptor availability, ligand binding, viral entry, or endothelial immune function are required to determine the biological relevance of these modest changes.

Several mechanisms may explain these findings. Similarly to other glucocorticoids, budesonide exerts its biological effects through glucocorticoid receptor-dependent regulation of inflammatory and immune pathways. The observed reduction in secreted IL-8 concentration and RANTES expression is consistent with the well-established anti-inflammatory activity of corticosteroids and likely reflects the attenuation of endothelial chemokine production, which may reduce leukocyte recruitment within the pulmonary microenvironment [7,8]. Although the decrease in IFN-β mRNA expression did not reach statistical significance in the present dataset, this observation is biologically plausible because corticosteroids are known to suppress type I interferon responses, which are central regulators of innate antiviral immunity [2,9]. Given that type I interferon signaling has been implicated in ACE2 regulation, this mechanism may partially explain the reduction in ACE2 mRNA levels observed following budesonide exposure [2].

One of the interesting observations of the present study was the discrepancy between ACE2 mRNA and surface protein expression. While budesonide reduced ACE2 mRNA expression, surface ACE2 expression was modestly increased at a later time point. This finding suggests that ACE2 regulation in pulmonary endothelial cells may involve mechanisms beyond direct changes in mRNA expression, including altered protein turnover, intracellular trafficking and post-transcriptional regulatory processes. This pattern appeared to be specific to ACE2, as AP-N and DPP4 showed concordant reductions at both the mRNA and protein levels. These observations suggest that the regulation of coronavirus-associated receptors in endothelial cells may differ depending on the receptor-specific regulatory mechanisms. Such discordance between mRNA and surface protein expression may reflect differences in protein turnover, surface retention, intracellular trafficking, or the timing of transcriptional and protein-level responses. Therefore, the modest increase in ACE2 surface expression should be interpreted cautiously and requires further validation using total protein analysis, intracellular staining, or assays assessing receptor trafficking and stability. Technical factors related to surface staining efficiency, epitope accessibility or flow-cytometric detection may also contribute to this discrepancy and should be considered when interpreting these modest surface changes.

The observed modulation of ICAM-1 expression also warrants consideration. ICAM-1 is a key endothelial adhesion molecule involved in leukocyte recruitment and vascular inflammatory activation [4]. In our model, budesonide reduced early ICAM-1 mRNA expression, whereas surface ICAM-1 expression was increased at 72 h. Although glucocorticoids are generally associated with the suppression of endothelial inflammatory activation, their effects under basal, non-inflammatory conditions may be more complex and influenced by timing, endothelial adaptation, and post-transcriptional regulatory mechanisms [10].

This study assessed the direct effects of budesonide on pulmonary endothelial cells in vitro. Nevertheless, these findings are relevant in the context of the growing interest in the broader immunomodulatory effects of inhaled corticosteroids in respiratory viral diseases, particularly COVID-19. Clinical studies have suggested that inhaled budesonide may improve outcomes in selected patients with early COVID-19, while observational studies have indicated that chronic inhaled corticosteroid therapy may influence the course of SARS-CoV-2 infection in patients with chronic airway diseases [11,12,13]. Although our model does not directly address infection outcomes, the observed modulation of coronavirus-associated receptor expression and basal antiviral mediator expression in pulmonary endothelial cells supports the concept that corticosteroid effects may extend beyond the airway epithelium and involve additional cellular compartments relevant to respiratory viral pathogenesis.

Our findings are consistent with those of previous studies demonstrating corticosteroid-mediated modulation of coronavirus-related pathways in airway epithelial models. Finney et al. reported that inhaled corticosteroids reduce ACE2 expression in patients with COPD by suppressing type I interferon signaling [2], while Peters et al. demonstrated altered expression of COVID-19-related genes in sputum cells from corticosteroid-treated patients with asthma [3]. In vitro studies have also shown that budesonide may suppress the replication of common respiratory viruses and modulate epithelial antiviral responses [14]. However, these studies focused primarily on epithelial cells, whereas our study specifically addressed the pulmonary microvascular endothelium. This distinction is important because endothelial cells differ substantially from epithelial cells in terms of immune function and receptor regulation. Our previous study demonstrated that pulmonary endothelial expression of ACE2, DPP4, and AP-N may be dynamically modulated by inflammatory stimuli [6]. The present findings extend these observations to the pulmonary microvascular endothelium by showing that budesonide alone, under basal non-stimulated conditions, alters endothelial receptor expression and selected immune-mediator expression.

The budesonide concentration used in the present study also requires consideration. The concentration of 1 ng/mL corresponds to approximately 2.3 nM budesonide and therefore represents a low nanomolar exposure. This concentration was selected to assess direct endothelial responses to budesonide under non-cytotoxic conditions while minimizing potential non-specific effects associated with higher corticosteroid exposure. Although the present study did not include a full dose–response analysis, the use of budesonide in respiratory structural-cell models is supported by previous in vitro work showing that budesonide can modulate respiratory virus-associated responses and cytokine production in airway epithelial cells [14]. Therefore, the selected concentration was considered appropriate for evaluating direct, basal endothelial responses to budesonide in the present mechanistic model.

This study has several limitations. First, this was an in vitro study performed using a commercially available HMVEC-L model derived from only two donors, which limits the ability to assess donor-to-donor variability. Moreover, cultured endothelial cells may not fully reproduce receptor expression patterns and regulatory mechanisms present in the intact pulmonary vasculature in vivo. Second, the molecular mechanisms underlying the observed receptor-specific effects were not directly investigated, and the proposed explanations remain hypothetical in nature. Third, IFN-β was assessed only at the mRNA level because reproducible protein-level results could not be established. Finally, the experimental model evaluated isolated endothelial cells under basal conditions and did not reproduce the complexity of multicellular interactions present within the respiratory microenvironment. Another limitation is that only one budesonide concentration was used in the main mechanistic experiments. Therefore, concentration-dependent effects could not be assessed and should be evaluated in future studies.

Despite these limitations, this study provides novel evidence that budesonide directly alters both receptor expression and basal immune-mediator expression inhuman lung microvascular endothelial cells. These findings expand the current understanding of inhaled corticosteroid biology beyond the airway epithelium and identify the pulmonary endothelium as a relevant target for corticosteroid-mediated immunomodulation.

Further studies are required to better understand the mechanisms underlying the differential effects of budesonide on endothelial receptor and immune mediator expression and to confirm these findings in experimental models that more closely reflect the complexity of the pulmonary microenvironment, including multicellular co-culture systems and in vivo models.

## 5. Conclusions

Budesonide directly modulates pulmonary endothelial immune responses and the expression of coronavirus-associated receptors in human lung microvascular endothelial cells. Specifically, budesonide reduced AP-N and DPP4 expression at both the mRNA and protein levels, transiently decreased ACE2 mRNA expression while modestly increasing ACE2 surface expression, and altered ICAM-1 expression in a time-dependent manner. In addition, budesonide modulated selected basal antiviral and inflammatory mediators, significantly reducing RANTES/CCL5 and IL-8/CXCL8 expression, while IFN-β mRNA expression showed a non-significant statistical decrease.

These findings indicate that the immunomodulatory effects of budesonide may extend beyond the airway epithelium and involve the pulmonary microvascular endothelium. The observed effects on coronavirus-associated receptors and endothelial immune-mediator expression provide new insights into the mechanisms through which inhaled corticosteroids may influence receptor and immune-mediator expression in the context of respiratory viral diseases. However, because viral binding, entry, replication, and infection were not assessed, the present study does not demonstrate altered susceptibility to coronavirus infection. Further studies are required to elucidate the molecular mechanisms underlying these effects and to determine their functional relevance in more complex experimental models, including functional viral infection models, and in vivo settings.

## Data Availability

The data supporting the findings of this study are available from the corresponding author upon reasonable request.

## Supplementary Materials

The following supporting information can be downloaded at: https://www.mdpi.com/article/10.3390/medicina62071347/s1, Table S1: Antibodies and corresponding isotype controls used for flow-cytometric analysis of surface receptor expression in HMVEC-L cells; Table S2: Raw and normalized MFI values used for flow-cytometric analysis of AP-N, DPP4, ACE2, and ICAM-1 surface expression in HMVEC-L cells; Table S3: Exact sample sizes and *p*-values for the comparisons shown in Figure 2 and Figure 3; Figure S1: Representative gating strategy for flow-cytometric analysis of DPP4 surface expression in HMVEC-L cells. (A) The main cell population was selected based on forward scatter (FSC-A) and side scatter (SSC-A), excluding debris and cell fragments. (B) Singlets were identified using FSC-A/FSC-H parameters. (C) Representative isotype control histogram. (D) Representative DPP4 staining histogram. The same gating strategy was applied to all analyzed surface markers.

## Abbreviations

The following abbreviations are used in this manuscript:

ACE2	Angiotensin-Converting Enzyme 2
AP-N	Aminopeptidase N
BSA	Bovine Serum Albumin
COPD	Chronic Obstructive Pulmonary Disease
DMSO	Dimethyl Sulfoxide
DPP4	Dipeptidyl Peptidase-4
EDTA	Ethylenediaminetetraacetic Acid
ELISA	Enzyme-Linked Immunosorbent Assay
GAPDH	Glyceraldehyde-3-Phosphate Dehydrogenase
HMVEC-L	Human Lung Microvascular Endothelial Cells
ICAM-1	Intercellular Adhesion Molecule 1
ICS	Inhaled Corticosteroids
IFN-β	Interferon Beta
IL-8/CXCL8	Interleukin-8
qRT-PCR	Quantitative Reverse Transcription Polymerase Chain Reaction
RANTES/CCL5	Regulated upon Activation, Normal T Cell Expressed and Secreted
RT-PCR	Reverse Transcription Polymerase Chain Reaction
SARS-CoV	Severe Acute Respiratory Syndrome Coronavirus
SARS-CoV-2	Severe Acute Respiratory Syndrome Coronavirus 2
SEM	Standard Error of the Mean
