# Supplementary material for: Effects of Budesonide on Coronavirus-Associated Receptor and Immune-Mediator Expression in Human Lung Microvascular Endothelial Cells
Source: Medicina (Kaunas). 2026 Jul 12;62(7):1347. doi: 10.3390/medicina62071347 (PMC13413501; doi:10.3390/medicina62071347)
Supplement: Supplementary file 1 [file medicina-62-01347-s001.zip › medicina-4405002-supplementary.pdf]

Supplementary Table S1. Antibodies and corresponding isotype controls used for flow-cytometric analysis of surface receptor expression in HMVEC-L cells.

Antibodies	Dilution	Isotype controls
**anti-ACE2 antibodies**<br>(Alexa Fluor 594 anti-human, FAB9332T, Novus Biologicals, Centennial, CO, USA)	1:200	Alexa Fluor-594 Mouse IgG_{2A}, κ isotype (IC003T, R&D Systems, Minneapolis, MN, USA) **for ACE2**
**anti-DPP4 antibodies**<br>(Alexa Fluor 594 anti-human CD26, FA1180T, Novus Biologicals, Centennial, CO, USA),	1:200	Alexa Fluor-594 Rat IgG_{2A}, κ isotype (IC006T, R&D Systems, Minneapolis, MN, USA)<br>**for DPP4**
**anti- AP-N antibodies**<br>(FITC, anti-human CD13, NBP2-47892F, Novus Biologicals, Centennial, CO, USA)	1:100	FITC Mouse IgG1, κ isotype (AB_11043278, Novus Biologicals, Centennial, CO, USA)<br>**for AP-N**
**anti-ICAM-1 antibodies**<br>(PE anti-human CD54, 322708, Biolegend, San Diego, CA, USA)	1:200	PE IgG1, κ isotype (400114, Biolegend, San Diego, CA, USA) **for ICAM-1**

Supplementary Table S2. Raw and normalized MFI values used for flow-cytometric analysis of AP-N, DPP4, ACE2, and ICAM-1 surface expression in HMVEC-L cells.

Marker	Independent experiment	Untreated control MFI	Budesonide MFI	Normalized MFI (BUD/Control)	Budesonide (% of control)
AP-N	1	446	392	0,879	87,9
AP-N	2	420	377	0,898	89,8
AP-N	3	445	391	0,879	87,9
AP-N	4	407	382	0,939	93,9
DPP4	1	461	454	0,985	98,5
DPP4	2	506	464	0,917	91,7
DPP4	3	493	474	0,961	96,1
DPP4	4	857	773	0,902	90,2
ACE2	1	150	154	1,027	102,7
ACE2	2	150	159	1,060	106,0
ACE2	3	176	183	1,040	104,0
ACE2	4	176	190	1,080	108,0
ICAM-1	1	150	165	1,100	110,0
ICAM-1	2	163	189	1,160	116,0
ICAM-1	3	177	185	1,045	104,5
ICAM-1	4	151	166	1,099	109,9

Each row represents one independent experiment. Normalized MFI was calculated as budesonide-treated MFI divided by untreated control MFI. Budesonide percentage of control was calculated as normalized MFI × 100. MFI, mean fluorescence intensity; BUD, budesonide.

Supplementary Table S3. Exact sample sizes and p-values for the comparisons shown in Figures 2 and 3.

Figure/Panel	Endpoint	n	Exact p-value
Fig. 2A	AP-N mRNA, 24 h	4	0.0079
Fig. 2A	AP-N mRNA, 72 h	5	0.0476
Fig. 2B	AP-N surface expression, 72 h	4	0.0286
Fig. 2C	DPP4 mRNA, 24 h	4	0.0079
Fig. 2C	DPP4 mRNA, 72 h	5	0.0079
Fig. 2D	DPP4 surface expression, 72 h	4	0.0286
Fig. 2E	ACE2 mRNA, 24 h	4	0.0079
Fig. 2E	ACE2 mRNA, 72 h	4	0.2063
Fig. 2F	ACE2 surface expression, 72 h	4	0.0286
Fig. 2G	ICAM-1 mRNA, 5 h	5	0.0476
Fig. 2G	ICAM-1 mRNA, 24 h	4	>0.9999
Fig. 2H	ICAM-1 surface expression, 72 h	4	0.0286
Fig. 3A	IFN-β mRNA, 5 h	4	0.3143
Fig. 3B	RANTES/CCL5 mRNA, 72 h	5	0.0079
Fig. 3C	IL-8/CXCL8 mRNA, 72 h	5	0.0079
Fig. 3D	Secreted IL-8 concentration, 72 h	5	0.0476

The table presents the exact number of independent experiments and exact p-values for each analyzed endpoint. Statistical comparisons between untreated control and budesonide-treated cells were performed using the Mann–Whitney U-test.

Representative gating strategy for flow-cytometric analysis of DPP4 surface expression in HMVEC-L cells

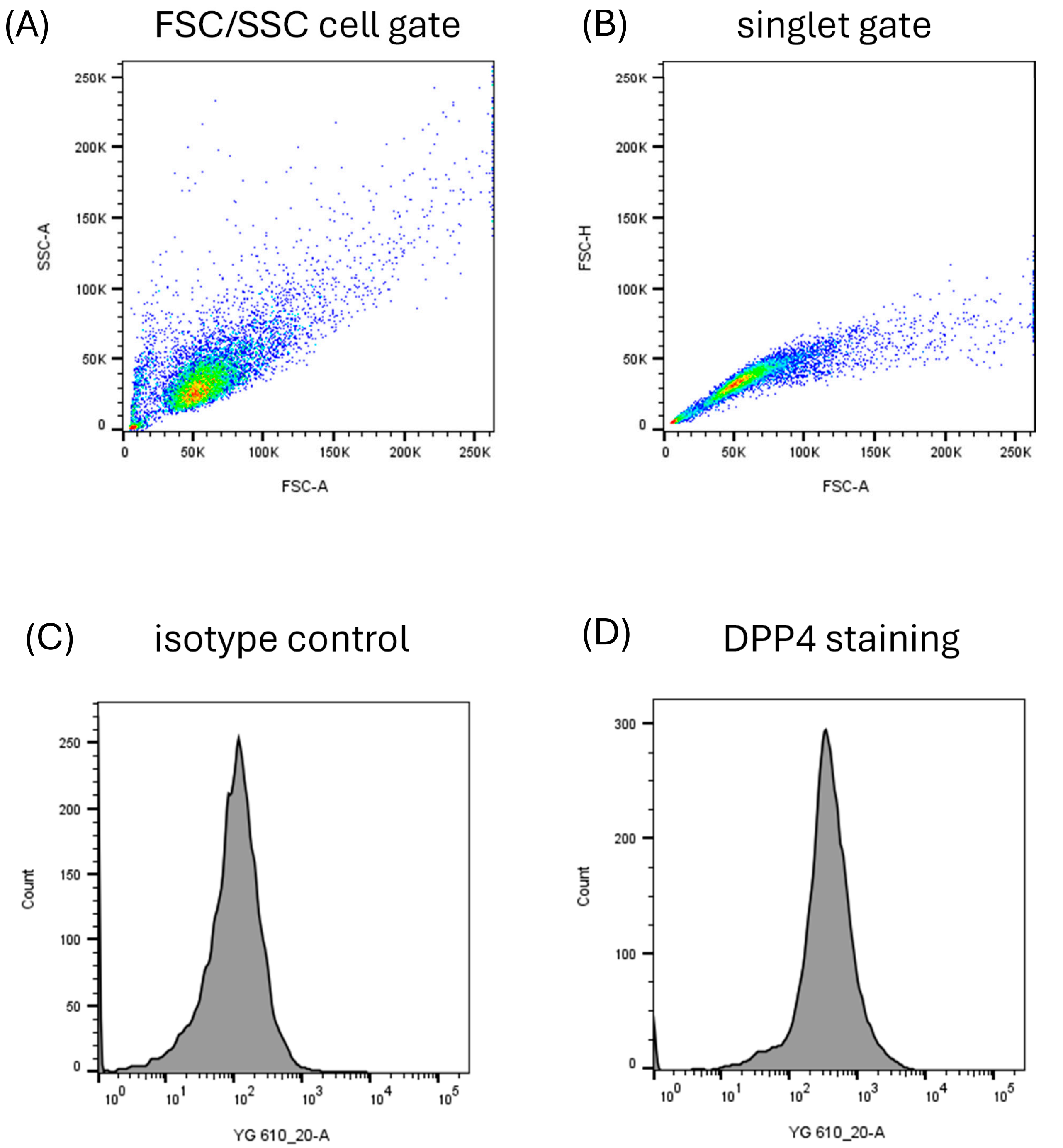

Supplementary Figure S1. Representative gating strategy for flow-cytometric analysis of DPP4 surface expression in HMVEC-L cells. (A) The main cell population was selected based on forward scatter (FSC-A) and side scatter (SSC-A), excluding debris and cell fragments. (B) Singlets were identified using FSC-A/FSC-H parameters. (C) Representative isotype control histogram. (D) Representative DPP4 staining histogram. The same gating strategy was applied to all analyzed surface markers.

Supp. Fig.1.
